# Incidence and Predictors of Structural Valve Deterioration after Transcatheter Aortic Valve Replacement: A Systematic Review and Meta-Analysis

**DOI:** 10.1155/2020/4075792

**Published:** 2020-11-04

**Authors:** Yu-Xiang Long, Zeng-Zhang Liu

**Affiliations:** Department of Cardiology, The Second Affiliated Hospital of Chongqing Medical University, Chongqing 400010, China

## Abstract

**Background:**

Transcatheter aortic valve replacement (TAVR), widely used as an alternative therapy in patients with severe aortic stenosis, is expected to be offered to low-risk patents with a longer life expectancy. The durability of transcatheter aortic valve is becoming of increasing importance.

**Method:**

PubMed, Embase, and Cochrane CENTRAL from the inception to March 2020 were systematically screened for studies reporting on structural valve deterioration (SVD) in TAVR patients. Incidence of SVD was diagnosed according to the latest European consensus as the primary end point. Predictors of SVD evaluated at multivariable analysis and cumulative incidence function (CIF) of SVD were the secondary end point.

**Result:**

Twelve studies encompassing 10031 patients evaluating the incidence of SVD were included, with a follow-up between 1 and 8 years. The pooled incidence of SVD was 4.93% (95% CI, 2.75%–7.70%, *I*^2^ = 96%) at 1 year and 8.97% (95% CI, 6.89%–11.29%, *I*^2^ = 86%) in the long term (≥5 years). Subgroup analysis was performed to identify the valve type that may result in partial heterogeneity. SVD was more frequent in patents with a valve diameter of <26 mm (HR: 3.57, 1.47–8.69), oral anticoagulants (OAC), exposure at discharge (OR: 0.48, 0.38–0.61), or by a disease of renal dysfunction (OR 1.42, 1.03–1.96).

**Conclusion:**

SVD represents infrequent events after TAVR in the long term (>5 years), occurring more commonly in renal dysfunction patients, with small valve diameter and without OAC exposure. There may be an underestimation of the incidence if we assume death as a competing risk.

## 1. Introduction

Transcatheter aortic valve replacement (TAVR), widely accepted as one of the major advances in therapy of severe aortic stenosis, was limited to high-risk or inoperable patients originally [1]. As the use of TAVR has grown rapidly, safety and efficacy has been demonstrated by accruing clinical evidences. Two randomized clinical trials (PARTNER 2 and SURTAVI) [2, 3] indicated that TAVR is noninferior to surgical aortic valve replacement (SAVR) in all-cause death and stroke within 2 years of follow-up. ESC guidelines in 2017 recommended to extend the indication to intermediate-risk patients [4]. Furthermore, there were studies focusing on TAVR in low-risk population, and they presented noninferior [5] or even superior [6] outcomes compared with SAVR. The anticipation of target patients in TAVR with a longer life expectancy has already raised concerns about the durability of transcatheter aortic valves (TAVs).

Structural valve deterioration (SVD), mainly the cause of bioprosthetic valve failure (BVF) in the long term, was reported as an infrequent event within the first 5 years after TAVR [7]. In consideration of the limited, long-term follow-up data and the lack of a consensus definition of SVD across studies, the pooled incidence of SVD was likely underestimated and doubted. A consensus statement from the European Association of Percutaneous Cardiovascular Interventions (EAPCI) was published in 2017, which applied a standardized definition of SVD for use in future studies [8].

In light of the definition of EAPCI, several studies have been published on SVD after TAVR, although the incidence, and the clinical and procedural predictors of SVD remain unclear. Uncertainty of the long-term (>5 years) durability of THVs creates doubts as to whether TAVR can be used in younger patients with a longer life expectancy. Therefore, we present a meta-analysis to clarify these issues and optimize the management of patients at risk.

## 2. Method

### 2.1. Search Strategy and Study Selection

Our work was conducted in compliance with the Preferred Reporting Items for Systematic Reviews and Meta-analysis (PRISMA). The review was also registered online at its inception on PROSPERO to minimize duplication.

We searched the Embase, PubMed, and Cochrane CENTRAL from the inception dates to March 2020, using the key words: (((Transcatheter aortic valve replacement) or (Transcatheter aortic valve implantation) or (TAVI) or (TAVR)) and ((Structural valve deterioration) or (Structural valvular deterioration) or (Structural valve degeneration) or (Structural valvular degeneration) or (SVD) or (Valve haemodynamic deterioration) or (VHD) or (valve deterioration))). There were no language restrictions, and the literature was searched by a single researcher. We also checked the bibliographies of potentially relevant studies and reviews for additional studies.

Two independent reviewers (Y.L. and Z.L.) screened the citations through the title and the abstract, and we made a mutual agreement procedure to reach a consensus if divergences existed. Studies that met the following explicit selection criteria were evaluated as complete reports:

1. Reporting the incidence of SVD and follow-up time in patients after TAVR on the basis of EAPCI definition strictly
2. Reporting the cumulative incidence function (CIF) of SVD assuming death as a competing risk
3. Reporting the predictors of SVD evaluated at multivariable analysis

The standard of exclusion criteria includes: case reports, case series, editorials, literature review, conference abstract, non-English articles, and duplicate reports (we selected the study reporting the largest sample of patients in these cases).

### 2.2. Data Abstraction and Risk of Bias Assessment

According to the consensus from EAPCI [8], the specific definition of structural valve deterioration (SVD) is presented as follows: Moderate SVD: (i) mean gradient ≥20 and <40 mmHg and/or ≥10 and <20 mmHg change from baseline (before discharge or within 30 days of valve implantation) and/or (ii) moderate, new, or worsening (>1+/4+) intra-prosthetic aortic regurgitation. Severe SVD: (i) mean gradient ≥40 mmHg and/or ≥20 mmHg change from baseline (before discharge or within 30 days of valve implantation) and/or (ii) severe new or worsening (>2+/4+) intra-prosthetic aortic regurgitation.

Incidences of SVD in patients undergoing TAVR were the primary end points, and CIF of SVD and predictors of SVD at multivariate analysis evaluated in each study were the secondary end points.

Two independent reviewers (Y.L. and Z.L.) extracted the data on prespecified forms: authors; year of publication; type of study; sample size; baseline characteristics, including age, sex, body mass index (BMI) (kg/m^2^), history of coronary artery disease (CAD), diabetes mellitus, hypertension, peripheral arterial disease, chronic obstructive pulmonary disease (COPD), pacemaker implantation, stroke, and myocardial infarction; New York Heart Association (NYHA), the European System for Cardiac Operative Risk Evaluation (EuroSCORE I), the Society of Thoracic Surgeons (STS) score and the left ventricular ejection fraction (LVEF); procedure features, including the TAV type, access routine, aortic valve area (cm^2^) at baseline, and mean transvalvular aortic gradient (mmHg) before/after operation. In instances where incomplete data were obtained, the authors were contacted in writing for permission to obtain further data.

For assessing the risk of bias in the included studies, we use the Quality in Prognostic Studies (QUIPS) tool, which includes questions related to areas that can inform judgments of risk of bias in prognostic research [9]. We make consensus to rate the included studies as involving low, moderate, or high risk of bias based on the following criteria: five or six low-risk domains as the overall low risk of bias, two or more high-risk domains as the overall high risk of bias, and the remaining studies as overall moderate risk of bias. We used the Grading of Recommendations, Assessment, Development, and Evaluation (GRADE) system to assess the quality of evidence for prognostic questions [10].

### 2.3. Statistical Analysis

Continuous variables are presented using mean and standard deviation or median and interquartile range. Categorical data are presented as percentages. Random-effect meta-analysis was performed because of the observational design of the included studies. Meta-analysis of incidence rates was performed using an arcsine transformation for the individual proportions, with the pooled proportion calculated as the back-transformation of the weighed mean of the transformed proportion. To identify the sources of heterogeneity, we performed subgroup analysis according to the principle that a study that performed 80% or more of the TAVR procedures with either valve was subcategorized to the respective group, and studies with less than 80% predominance of either valve were subcategorized to Mixed group. Funnel plot analysis with the Egger's test was used to evaluate potential publication bias, and the *I*^2^ test was used to investigate heterogeneity. For meta-regression, we exploited the “meta” packages in *R* studio software, which is a function to fit the meta-analytic random-effects models with moderators via linear (mixed-effects) models. When reporting the independent predictors of SVD, we extracted the adjusted odd ratios (ORs), or hazard risks (HRs), and their 95% confidence interval (CI) to compute a pooled OR/HR with 95% CI. Meta-analysis of OR/HR was performed after logarithmic transformation, and the results with the corresponding 95% CI were reported after back-transformation. Statistical analyses were performed with STATA 14, *R* Studio 3.6.3, and SPSS Version 25.

## 3. Results

**Figure 1:**
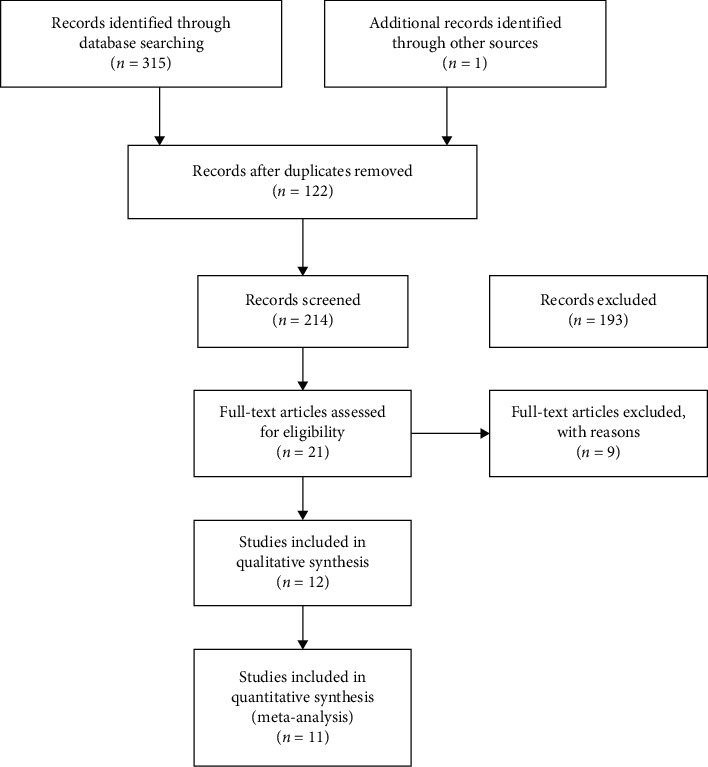
Flow diagram of search strategy and study selection.

Two-hundred and fourteen studies were identified by the electronic database searches after removing duplicate articles, of which 202 did not meet the inclusion criteria for reasons such as not reporting the incidence of SVD or not following the guideline of EAPCI. Ultimately, 12 studies [11–22] (observational studies), encompassing 10031 patients, were included according to the selection/exclusion criteria (Figure 1). Their mean age was 82.4 years. 50.7% of the patients were men, and 53.8% of the patients were implanted with an Edwards valve. Clinical features of the patients in the included studies are summarized in Table 1 and Supplementary Table 1. Assuming death as a competing risk, 3 studies reported the CIF of SVD [11, 12, 16]. Incidence at 1 year and 5 years or more from included studies were pooled in meta-analyses, respectively. Except one article, the others reported the incidence of SVD at 3.2 years [19]. Two studies were rated as moderate risk of bias through QUIPS risk assessment [14, 17], and the overall quality of evidence was rated down to moderate due to inconsistency (Supplementary Tables 2 and 3) [14, 17].

**Figure 2:**
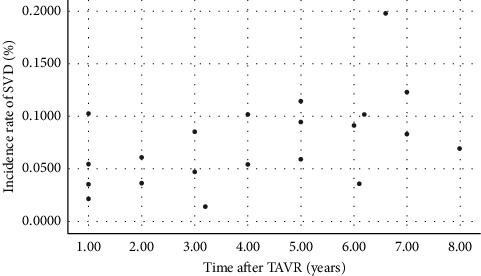
Incidence rate of SVD across all studies. SVD: structural valve deterioration; TAVR: transcatheter aortic valve replacement.

The incidence of SVD was reported to range from 3.1% in French [13] to 10.3% in German [21], by 1 year; from 3.6% in Italy [18] to 19.8% in German [22], by 5 years or more.  Figure 2 shows the incidence of SVD across all the included studies (specific data are presented in Supplementary Table 4).

**Figure 3:**
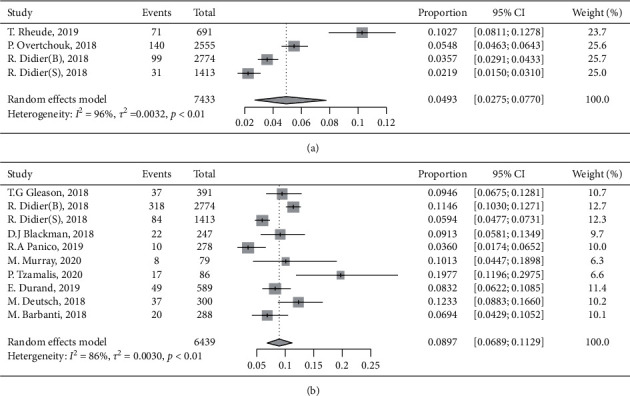
Forest plots of structural valve deterioration (SVD) incidence rate for (a) short term (1 year after TAVR) and (b) long term (≥5 years). CI: confidence interval.

**Figure 4:**
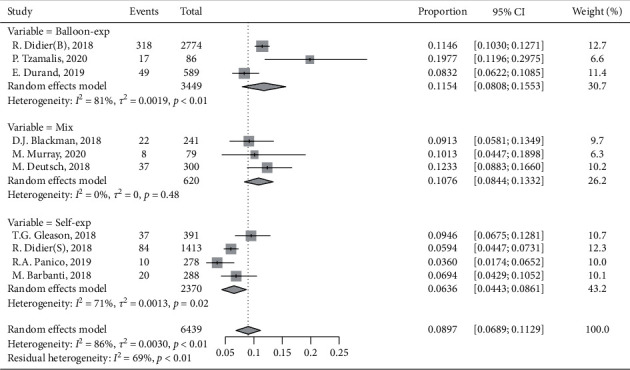
Subgroup analysis on valve type. Balloon-exp: balloon-expandable valve; Self-exp: self-expandable valve.

According to the various times of follow-up between the studies, we classified the included studies into short-term (1 year) or long-term groups (5 years or more) and pooled the SVD incidence rate accordingly. The pooled incidence of SVD was 4.93% (95% CI, 2.75%–7.70%, *I*^2^ = 96%) in the short-term group (Figure 3(a)) and 8.97% (95% CI, 6.89%–11.29%, *I*^2^ = 86%) in the long-term group (Figure 3(b)). We performed the funnel plot and Egger's test, which indicated no publication bias in the long-term group (Supplementary Figures 1 and 2). We performed subgroup analysis to explore the confounders due to high heterogeneity across the studies (Figure 4). It suggested that the difference in valve type across the studies may account for part of heterogeneity. Moreover, a higher incidence rate of SVD was seen with an increasing use of balloon-expandable valve, but it did not reach a significant difference. Then, the assessment of potential moderator variables through meta-regression revealed significant associations between a balloon-expandable valve and a higher incidence of SVD, and no other associations were seen (Supplementary Figures 3 and 5). However, multivariate analysis results from two included studies were pooled, which indicated that a balloon-expandable valve was not a predictor of SVD (HR: 1.03, 95%: 0.34 to 3.17).

**Figure 5:**
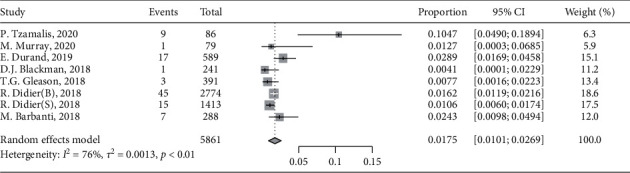
Forest plots of severe structural valve deterioration incidence rate for the long term (≥5 years).

Seven included studies reported long-term incidence of moderate or severe SVD, respectively, and the pooled incidence of severe SVD was 1.75% (95%: 1.01 to 2.69, *I*^2^ = 76%) (Figure 5).

The CIF of SVD was reported to range from 10.8% (95% CI, 7.59 to 14.63) in Durand et al. [16] to 14.9% (95% CI, 10.67 to 19.10) in Deutsch et al. [12] at 7 years, and the CIF of severe SVD was reported to range from 2.39% (95% CI, 0.77 to 5.36) in Barbanti et al. [11] at 8 years to 4.2% (95% CI, 1.98 to 7.43) in Durand et al. [16] at 7 years. With limited data, we did not perform a meta-analysis on the CIF of SVD.

**Figure 6:**
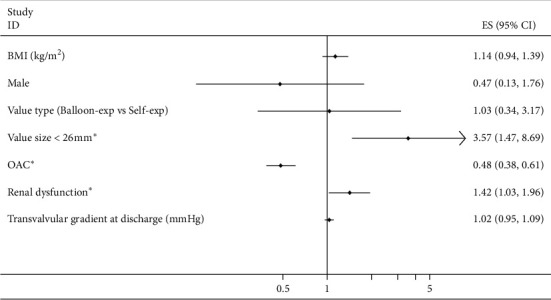
Independent predictors of structural valve deterioration in the included studies after multivariate analysis. BMI: body mass index; OAC: oral anticoagulant. ^*∗*^Significant difference.

The independent predictors of SVD in the included studies (Figure 6) were: small valve diameter (<26 mm), renal dysfunction, and without OAC exposure at discharge.

## 4. Discussion

In this meta-analysis, we have discovered the following major outcomes:

1. SVD is infrequent in 5 years after TAVR, and severe cases are rare.
2. Patients with small valve diameter, renal dysfunction, or without OAC exposure at discharge are at an increased risk of SVD.
3. There is an association of balloon-expandable valve and SVD, and further studies are needed.

Incidence rate after TAVR. Due to the inconsistent definition of SVD across studies, the impact of SVD after TAVR has yielded conflicting results. Previous meta-analysis [7] has reported that SVD incidence ranged from 0 to 1.34% per year, and that the pooled incidence is 0.28% per year. Limited by short follow-up duration (SVD did not occur in seven of the included studies) and inconsistent definition, the true incidence of SVD may be underestimated.

Based on the standard definition of EAPCI, our work identified that SVD was associated with a hazard of 4.93% at 1 year and 8.97% in the long term (≥5 years), and the conclusions are stable when studies are removed from the analysis set one at a time. In spite of the low risk of bias assessed by the QUIPS tool, we rated the overall quality of evidence as moderate using the GRADE system due to inconsistency between the studies. In addition to valve types, this discrepancy in outcomes may also be related to the challenges in the identification and quantification of post-TAVR aortic regurgitation and the completion of follow-up.

Among patients diagnosed with SVD, most of them had moderate SVD, and the trend did not reverse with the follow-up going on. Therefore, in spite of a higher SVD incidence being presented, the incidence of severe SVD we pooled was extremely low (1.75%, 95% CI: 1.01 to 2.69). As the main population with moderate SVD was asymptomatic [11], fewer patients were confronted with re-intervention. The longest follow-up duration in our meta-analysis is 8 years, and our analysis suggests a reliable function of TAVs for at least 5 years. It may be an implication of expanding the indication to low-risk patients.

However, since patients included in our work were elderly and either inoperable or at a high risk for surgery, high mortality was observed in the major included studies. In accordance with the suggestion of the European consensus [8], three studies reported the CIF of SVD while assuming death as a competing risk [11, 12, 16], which is slightly higher than that we diagnosed. We consider that a better evaluation was performed when using the method of competing risk, but there is still uncertainty due to little evidence we included.

### 4.1. Valve type

Regardless of the valve type, post-TAVR SVD was not a common event in the long term. Nevertheless, we found a correlation between a higher SVD incidence and the implantation of balloon-expandable valve. Sellers et al. [23] suggested a sequential cascade of thrombus formation, fibrosis, and calcification, which ultimately contributes to progressive leaflet thickening and SVD. The balloon-expandable valve was demonstrated to be a predictor of leaf thrombosis [24], which may raise concerns about its long-term durability. Deutsch et al. [20] reported that the overall crude cumulative incidence of SVD at 7 years was 14.9% (CoreValve 11.8% vs Sapien 22.6%; *p*=0.01). However, with multivariate regression analysis in several studies [15, 16], there is no evidence to prove the connection of SVD and the valve type.

The reason for this finding is still unclear and there are several explanations in this regard. First, the different deployments between the two types of valves, balloon-expandable valve placed in an intra-annular position and a self-expandable valve placed in a supra-annular position, may introduce bias in measurement and cause heterogeneity; Second, tissue fissuring and endothelial denudation resulting from balloon-dilatation may increase procoagulant activity locally [24]. Finally, sustained expansion of the nitinol frame of the self-expanding device may reduce valve distortion and turbulence in the long term, which can affect hemodynamics and lower the risk of SVD [25].

TAVs used in included studies are mainly first-generation devices in an early stage of TAVR. Hence, second- and third-generation devices of both self-expandable and balloon-expandable valves are in routine use with a good performance in improving the periprocedural complications [26, 27]. Further intensive study is needed to explore the durability of various TAVs in the long term.

### 4.2. Predictive factors

SVD was less frequently observed in patients treated with OAC at discharge and more frequent in patients with renal dysfunction or in those using a small valve size (<26 mm). Reduction of the incidence of leaflet thrombosis (LT) with OAC, which also decreases the mean transvalvular gradient, was reported in a meta-analysis [24]. It may suggest that OAC can prevent SVD by reducing LT. Regarding high-risk TAVR patients, post-TAVR OAC exposure and OAC crossover remained significantly related to increased mortality [17, 28]. So far, there has been no strong evidence to identify this detrimental effect. Several causes may lead to this phenomenon, such as more comorbidities in patients taking OAC, new-onset atrial fibrillation after TAVR, and poor international normalized ratio (INR) management with vitamin K-based regimens. Meanwhile, due to short follow-up duration in the included studies (only 1 year), the long-term efficacy of OAC in preventing SVD has not been shown, and whether the long-term outcome would be different if novel oral anticoagulant-based strategies were employed in low-risk patients remains to be clarified.

In addition, the post-TAVR management of renal insufficiency is pretty tough. A retrospective study [29] reported a higher incidence of re-intervention in dialysis patients undergoing TAVR. Moreover, Sellers et al. [23] rightly note that patients undergoing TAVR with end-stage renal disease are at a higher risk with higher in-hospital mortality. These findings may have implications in deciding whether to send a dialysis patient for TAVR. Finally, in view of the association between small valve size and SVD, the histological analysis of Sellers et al. identified a time-dependent deterioration of TAVs consisting of thrombus formation in which 91.3% of the sample valves are smaller than or equal to 26 mm. Therefore, a patent-specific anticoagulant therapy for patients with a small valve size needs to be considered.

### 4.3. Limitation

A major limitation of this review is that all studies included were observational and significant heterogeneity was seen across studies for all the outcomes analyzed, thereby introducing limitation in the quality of data. Although we abided by the EAPCI definition of SVD strictly to keep the results homologous, this criterion may also lead to miss-selection of high-quality studies and introduce selection bias. Meta-analysis was performed on the basis of two time-points we selected; hence, our work did not reflect a continuous process well and the results will not be applied to other time-points. Limitations in reporting of clinical and procedural features prevented us from exploring subgroup hypotheses and multivariate regression. The main subjects in the included studies are the elderly with high- or intermediate-risk patients, and our work can only be used as an analogy material with caution for younger or low-risk patients.

## 5. Conclusion

Following the guideline of the European consensus, the incidence rate of SVD is infrequent at 5 years after TAVR. Due to the high mortality of subjects, there may be some underestimation when assuming death as a competing risk. The benefits of the self-expandable valve compared with the balloon-expandable valve in SVD are still not very clear and more powerful evidences are needed.

## Figures and Tables

**Table 1:** Baseline demographic and population characteristics.

Author	Follow-up (years)	Numbers	Age (year)	Male (%)	Log Euroscore (%)	STS score (%)	Valve type	Echocardiograph	Multicenter	Study design
Tzamalis [22]	6.6	86	78.3 ± 5.2	46.30	8.7 ± 2.7	NR	Sapien (37.5%), Sapien XT (43.5%), CoreValve (16.7%), Accurate (1.4%), JenaValve (1.3%)	Unclear	No	Observational; retrospective

Rheude [21]	1	691	80.0 ± 6.2	57.90	14.1 ± 10.0	NR	Sapien 3 (100%)	Baseline, discharge, once during the12-month follow-up after TAVR and yearly thereafter	No	Observational; retrospective

Murray [20]	6.2	79	80.1 ± 7.9	43.70	18.1 ± 11.7	9.1 ± 5.6	Sapien (58.8%), CoreValve (40.2%), JenaValve (1.0%)	Baseline and the late follow-up	No	Observational; prospective

Kefer [19]	3.2	346	84.0 ± 7.0	46.80	NR	6.7 ± 5.0	CoreValve (5.8%), Portico (8.9%), Evolut-R (34.4%), Sapien (10.1%), Sapien XT (34.7%), Sapien 3 (6.1%)	Unclear	No	Observational; prospective

Panico [18]	7	278	82.3 ± 5.5	47.50	19.7 ± 12.6	6.4 ± 5.0	CoreValve (100%)	Discharge, one month, six months, and yearly thereafter	No	Observational; prospective

Overtchouk [17]	1	2555	82.8 ± 0.1	49.60	17.8 ± 0.1	NR	Balloon-expandable valve (64.7%), self-expandable valve (35.3%)	Baseline, 1 month, 6 months, and annually	Yes	Observational; retrospective

Durand [16]	7	598	82.6 ± 7.5	51.80	21.3 ± 7.5	NR	Sapien (83.6%), CoreValve (15.7), and JenaValve (0.6%)	Discharge, 1 month and at the last follow-up visit	Yes	Observational; prospective

Blackman [15]	6	241	79.3 ± 7.5	53.90	19.7 ± 12.3	NR	Sapien (19.3%), Sapien XT (15.0%), CoreValve (64%), Portico (1.7%)	The most recent transthoracic echocardiogram, no less than 4 years 6 months post-TAVR	Yes	Observational; retrospective

Gleason [14]	5	391	83.2 ± 7.1	52.90	NR	7.3 ± 3.0	CoreValve (100%)	Discharge, 1 month, 6 months, and 1 year after the procedure	Yes	Observational; prospective

Didier (B) [13]	5	2774	83.0 ± 7.1	47.70	21.8 ± 14.1	NR	Sapien or Sapien XT (100%)	Baseline, 1 month, 6 months, and 1, 2, 3, 4, and 5 years.	Yes	Observational; prospective

Didier (S) [13]	5	1413	82.5 ± 7.3	60.30	21.5 ± 14.5	NR	CoreValve (100%)	Baseline, 1 month, 6 months, and 1, 2, 3, 4, and 5 years.	Yes	Observational; prospective

Deutsch [12]	7	300	81.4 ± 6.6	36.70	21.2 ± 13.3	6.5 ± 4.5	Sapien (28.7%), CoreValve (71.3%)	Discharge, 6 months and yearly thereafter	No	Observational; retrospective

Barbanti [11]	8	288	80.7 ± 5.3	41.70	NR	8.1 ± 5.1	Sapien XT (16.8%), CoreValve (82.6%)	Discharge, 1 and 12 months and then yearly after TAVR	No	Observational; prospective

Didier et al. (B) [13]: the group of patients using balloon-expandable valve; Didier et al. (S) [13]: the group of patients using self-expandable valve; NR: not reported in the study; Log Euroscore: logistic European System for Cardiac Operative Risk Evaluation; STS score: Society of Thoracic Surgeons score.

## Data Availability

The data supporting this systematic review and meta-analysis are from previously reported studies and datasets, which have been cited. The processed data are available from the corresponding author upon request.

## Supplementary Materials

Supplementary Materials Supplementary Table 1: baseline clinical characteristics of the included population. Supplementary Table 2: QUIPS risk of bias in individual studies. Supplementary Table 3: quality of evidence. Supplementary Table 4: specific value about the incidence rate of SVD after TAVR. Supplementary Table 5: meta-regression data. Supplementary Figure 1: funnel plots of structural valve deterioration (SVD) incidence rate for the long term. Supplementary Figure 2: Egger's test of structural valve deterioration (SVD) incidence rate for the long term. Supplementary Figure 3: meta-regression plot assessing the association of the percentage of balloon-expandable valve with the incidence of structural valve deterioration (SVD), compared with the self-expandable valve in the long term.
