# Supplementary material for: Incidence and Predictors of Structural Valve Deterioration after Transcatheter Aortic Valve Replacement: A Systematic Review and Meta-Analysis
Source: J Interv Cardiol. 2020 Nov 4;2020:4075792. doi: 10.1155/2020/4075792 (PMC7657687; doi:10.1155/2020/4075792)

**Supplementary materials:**

**Supplementary Table 1:** Baseline clinical characteristics of included population

| Author | Recruitment Period | Numbers | Diabetes mellitus (%) | CAD (%) | BMI (Kg/m^2^) | COPD (%) | Pacemaker (%) | Atrial fibrillation (%) | Ejection fraction * | Recent myocardial infraction (%) | Transvalvular aortic gradient at baseline (mmHg) | Femoral access (%) | Transvalvular aortic gradient at discharge (mmHg) | |
| --- | --- | --- | --- | --- | --- | --- | --- | --- | --- | --- | --- | --- | --- | --- |
| P. Tzamalis, 2020 | 2008-2012 | 86 | NR | 48.1 | NR | NR | NR | NR | 62.2±11.3 | 2.3 | NR | 62.8 | | 9.1±3.8 |
| T. Rheude,2020 | 2014-2018 | 691 | 29.8 | 72.5 | 27.0±4.7 | 14.0 | 11.9 | 42.1 | <35%, 11.6% | 11.1% | 43 [34-51] | 100.0 | | NR |
| M. Murray, 2020 | 2006-2012 | 79 | 22.3 | NR | 27.5±4.3 | 14.7 | 15.5 | 35.0 | NR | 18.4% | 51.8±14.3 | 65.0 | | 11.7±5.8 |
| J. Kefer,2019 | 2008-2018 | 346 | 18.0 | 60.0 | 26.0±5.0 | 27.0 | 19.0 | 38.0 | 56.0±15.0 | 28.0% | 45.0±15.0 | 100.0 | | NR |
| R.A. Panico, 2019 | 2007-2013 | 278 | 30.2 | 46.8 | 26.0±4.4 | 16.6 | 35.3 | NR | <50%, 37.4% | 14.4% | 52.0±16.0 | 78.4 | | 9.0±5.0 |
| P. Overtchouk, 2018 | 2013-2015 | 2555 | 26.0 | NR | 26.7±0.1 | NR | NR | 33.0 | 55.4±0.1 | NR | NR | 83.5 | | NR |
| E. Durand, 2019 | 2002-2011 | 598 | 24.0 | 48.3 | NR | NR | 14.9 | 32.4 | 52.8±14.4 | 18.9% | 48.1±17.0 | 65.4 | | NR |
| D.J. Blackman, 2018 | 2007-2011 | 241 | 23.3 | NR | NR | NR | NR | 25.0 | <50%, 30.3% | 25.6% | NR | 80.3 | | NR |
| T.G. Gleason, 2018 | 2011-2012 | 391 | 34.8 | 75.4 | NR | 44.8 | 23.3 | 41.0 | 56.9±12.5 | NR | NR | NR | | 7.1±3.6 |
| R. Didier(B),2018 | 2010-2012 | 2774 | 25.3 | 48.3 | 25.9±4.8 | 24.3 | 13.4 | 25.2 | 54.0±14.0 | 16.2 | 48.6±16.6 | NR | | 11.1±4.8 |
| R. Didier(S), 2018 | 2010-2012 | 1413 | 25.7 | 46.6 | 26.3±5.2 | 24.9 | 16.5 | 28.8 | 52.0±14.0 | 16.2 | 47.0±16.2 | NR | | 9.9±5.4 |
| M. Deutsch, 2018 | 2007-2009 | 300 | NR | 52.7 | 26.4±4.6 | 22.3 | 10.0 | 23.7 | <50%, 40.0% | NR | 48.6±16.9 | 65.7 | | 11.8±4.0 |
| M. Barbanti, 2018 | 2007-2012 | 288 | 26.4 | NR | 26.7±5.3 | 35.4 | 9.7 | 15.3 | 51.5±10.5 | 18.4 | 53.3±15.9 | 98.3 | | 10.5±4.5 |

*: Ejection fraction value are presented as mean ± standard deviation or percentage; CAD: coronary arterial diseases; BMI: body mass index.

**Supplementary Table 2**: QUIPS risk of bias in individual studies

| Study | Study Participation | Study Attrition | Prognostic Factor Measurement | Outcome Measurement | Study Confounding | Statistical Analysis and Reporting | Overall Risk of Bias |
| --- | --- | --- | --- | --- | --- | --- | --- |
| P. Tzamalis, 2020  T. Rheude,2020  M. Murray, 2020  J. Kefer,2019  R.A. Panico, 2019  P. Overtchouk, 2018  E. Durand, 2019  D.J. Blackman, 2018  T.G. Gleason, 2018  R. Didier(B),2018  R. Didier(S), 2018  M. Deutsch, 2018  M. Barbanti, 2018 | Low  Low  Low  Low  Low  Low  Low  Low  Low  Low  Low  Low  Low | Moderate  Low  Low  Moderate  High  Moderate  Moderate  Low  Moderate  Low  Low  Moderate  Low | Low  Low  Low  Low  Low  Low  Low  Low  Low  Low  Low  Low  Low | Low  Low  Low  Low  Low  Moderate  Low  Moderate  Moderate  Moderate  Moderate  Low  Low | Low  Low  Low  Low  Low  Low  Low  Low  Low  Low  Low  Low  Low | Low  Low  Low  Low  Low  Low  Low  Low  Low  Low  Low  Low  Low | Low  Low  Low  Low  Low  Moderate  Low  Low  Moderate  Low  Low  Low  Low |

| Supplementary Table 3: Quality of evidence | | | | | | | | |
| --- | --- | --- | --- | --- | --- | --- | --- | --- |
| Number of studies | **Quality assessment** | | | | | | **Effect** |  |
|  | **study design** | **Risk of bias** | **Inconsistency** | **Imprecision** | **Indirectness** | **Publication bias** | **Rate (95%CI)** | **Quality** |
| 3 | **Observational** | **Low** | **Serious** | **Not serious** | **Not serious** | **Not serious** | **4.93% (2.75%-7.70%)** | **Moderate** |
| 9 | **Observational** | **Low** | **Serious** | **Not serious** | **Not serious** | **Not serious** | **8.97% (6.89%-11.30%)** | **Moderate** |

**Supplementary Table 4:** Specific value about incidence rate of SVD after TAVR

| Study | Follow-up (year) | Numbers | Events | Incidence rate of SVD |
| --- | --- | --- | --- | --- |
| T. Rheude,2020 | 1.0 | 691 | 71 | 0.1027 |
| P. Overtchouk, 2018 | 1.0 | 2555 | 140 | 0.0548 |
| R. Didier(B),2018 | 1.0 | 2774 | 99 | 0.0356 |
| R. Didier(S), 2018 | 1.0 | 1413 | 31 | 0.0219 |
| R. Didier(B),2018 | 2.0 | 2774 | 169 | 0.0609 |
| R. Didier(S), 2018 | 2.0 | 1413 | 52 | 0.0368 |
| R. Didier(B),2018 | 3.0 | 2774 | 237 | 0.0854 |
| R. Didier(S), 2018 | 3.0 | 1413 | 67 | 0.0474 |
| J. Kefer,2019 | 3.2 | 346 | 5 | 0.0145 |
| R. Didier(B),2018 | 4.0 | 2774 | 283 | 0.1020 |
| R. Didier(S), 2018 | 4.0 | 1413 | 77 | 0.0545 |
| T.G. Gleason, 2018 | 5.0 | 391 | 37 | 0.0946 |
| R. Didier(B),2018 | 5.0 | 2774 | 318 | 0.1146 |
| R. Didier(S), 2018 | 5.0 | 1413 | 84 | 0.0594 |
| D.J. Blackman, 2018 | 6.0 | 241 | 22 | 0.0913 |
| R.A. Panico, 2019 | 7.0 | 278 | 10 | 0.0324 |
| M. Murray, 2020 | 6.2 | 79 | 8 | 0.1013 |
| P. Tzamalis, 2020 | 6.6 | 86 | 17 | 0.1977 |
| E. Durand, 2019 | 7.0 | 589 | 49 | 0.0832 |
| M. Deutsch, 2018 | 7.0 | 300 | 37 | 0.1233 |
| M. Barbanti, 2018 | 8.0 | 288 | 20 | 0.0694 |

**Supplementary Table 5:** Meta-regression data

| Variables | r | p value |
| --- | --- | --- |
| Age | -0.019 | 0.174 |
| % of male gender | -0.328 | 0.221 |
| % of balloon-expandable valve | 0.095 | **0.018** |
| Follow up duration | -0.007 | 0.741 |

**Supplementary Figure 1:** Funnel plots of structural valve deterioration (SVD) incidence rate for long-term


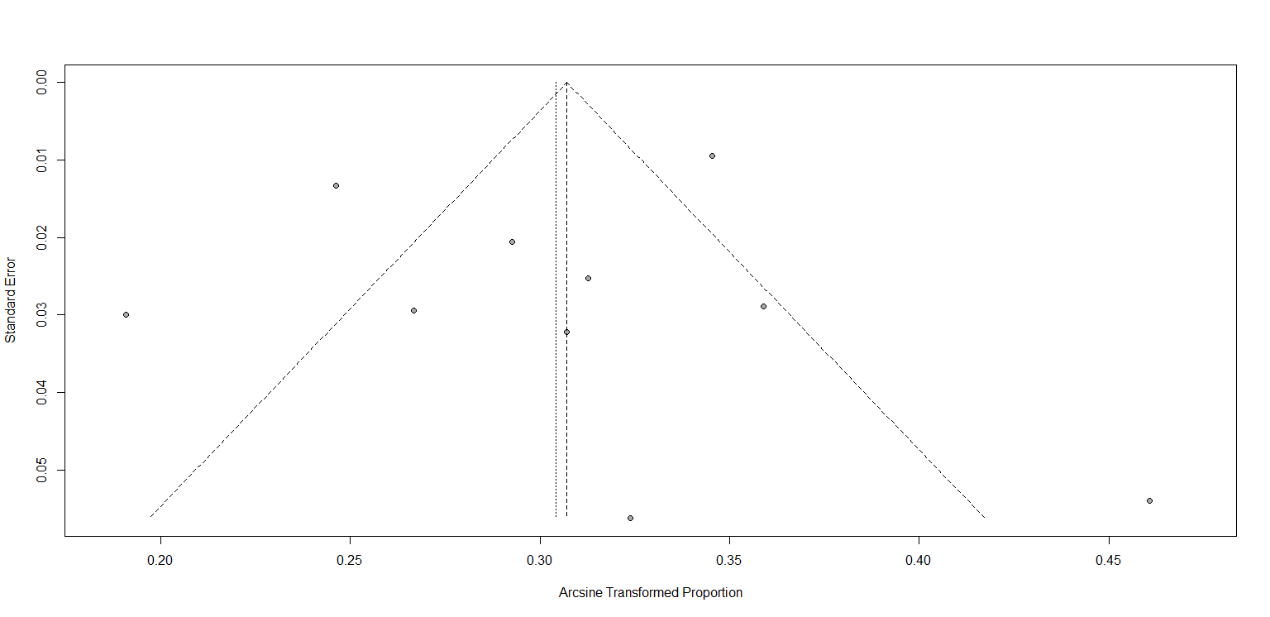


**Supplementary Figure 2:** Egger’s test of structural valve deterioration (SVD) incidence rate for long-term

**
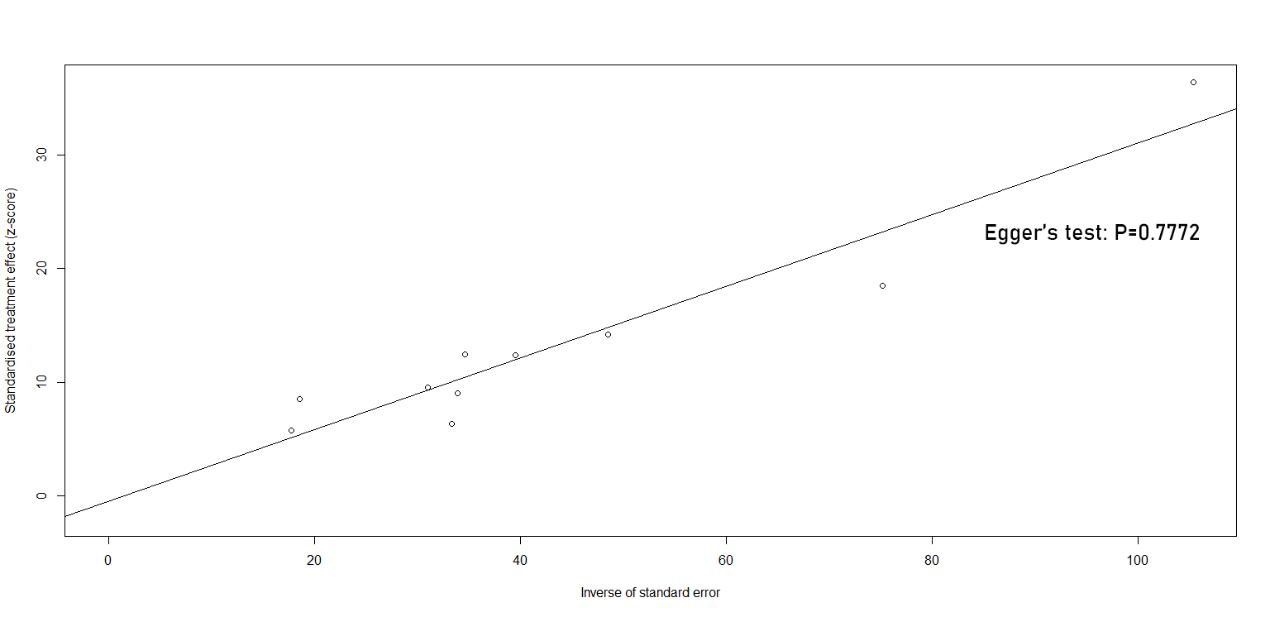
**

**Supplementary Figure 3:** Meta-regression plot assessing the association of percentage of balloon-expandable valve with the incidence of structural valve deterioration (SVD) compared with self-expandable valve in long-term


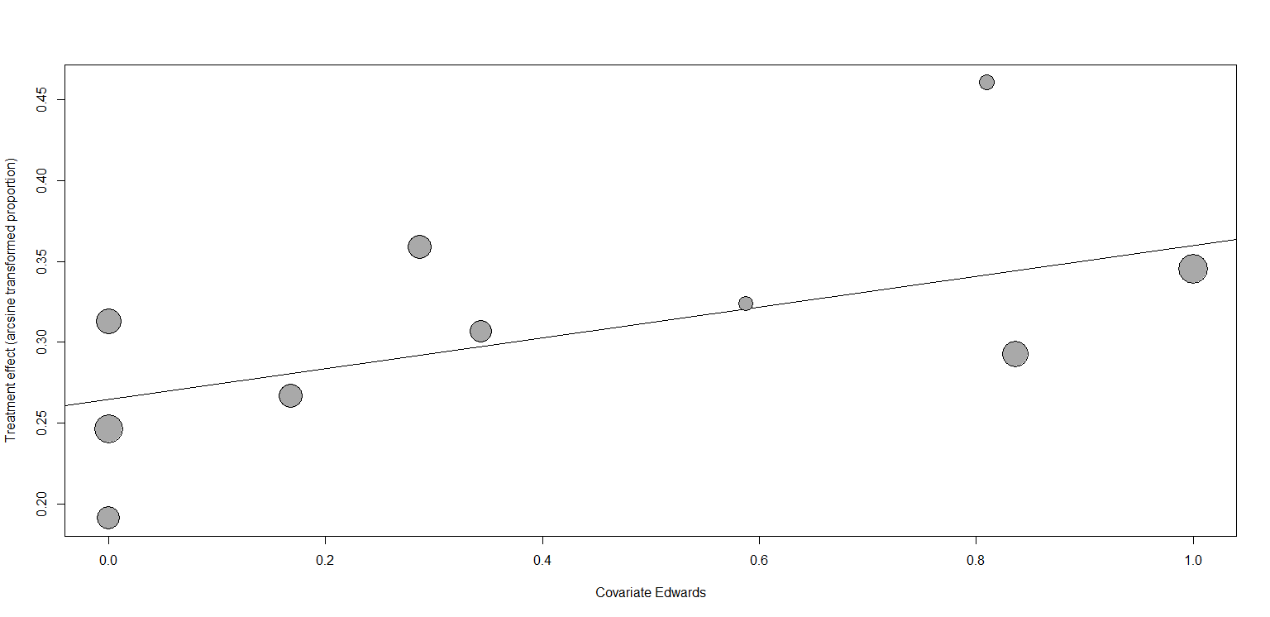

Supplement: Supplementary Materials — Supplementary Table 1: baseline clinical characteristics of the included population. Supplementary Table 2: QUIPS risk of bias in individual studies. Supplementary Table 3: quality of evidence. Supplementary Table 4: specific value about the incidence rate of SVD after TAVR. Supplementary Table 5: meta-regression data. Supplementary Figure 1: funnel plots of structural valve deterioration (SVD) incidence rate for the long term. Supplementary Figure 2: Egger's test of structural valve deterioration (SVD) incidence rate for the long term. Supplementary Figure 3: meta-regression plot assessing the association of the percentage of balloon-expandable valve with the incidence of structural valve deterioration (SVD), compared with the self-expandable valve in the long term. [file 4075792.f1.docx]
